# Characterization of Sigma 1 Receptor Antagonist CM-304 and Its Analog, AZ-66: Novel Therapeutics Against Allodynia and Induced Pain

**DOI:** 10.3389/fphar.2019.00678

**Published:** 2019-06-14

**Authors:** Thomas J. Cirino, Shainnel O. Eans, Jessica M. Medina, Lisa L. Wilson, Marco Mottinelli, Sebastiano Intagliata, Christopher R. McCurdy, Jay P. McLaughlin

**Affiliations:** ^1^Department of Pharmacodynamics, University of Florida, Gainesville, FL, United States; ^2^Department of Medicinal Chemistry, University of Florida, Gainesville, FL, United States

**Keywords:** Sigma, allodynia, analgesia, chronic pain, sedation, addiction

## Abstract

Sigma-1 receptors (S1R) and sigma-2 receptors (S2R) may modulate nociception without the liabilities of opioids, offering a promising therapeutic target to treat pain. The purpose of this study was to investigate the *in vivo* analgesic and anti-allodynic activity of two novel sigma receptor antagonists, the S1R-selective CM-304 and its analog the non-selective S1R/S2R antagonist AZ-66. Inhibition of thermal, induced chemical or inflammatory pain, as well as the allodynia resulting from chronic nerve constriction injury (CCI) and cisplatin exposure as models of neuropathic pain were assessed in male mice. Both sigma receptor antagonists dose-dependently (10–45 mg/kg, i.p.) reduced allodynia in the CCI and cisplatin neuropathic pain models, equivalent at the higher dose to the effect of the control analgesic gabapentin (50 mg/kg, i.p.), although AZ-66 demonstrated a much longer duration of action. Both CM-304 and AZ-66 produced antinociception in the writhing test [0.48 (0.09–1.82) and 2.31 (1.02–4.81) mg/kg, i.p., respectively] equivalent to morphine [1.75 (0.31–7.55) mg/kg, i.p.]. Likewise, pretreatment (i.p.) with either sigma-receptor antagonist dose-dependently produced antinociception in the formalin paw assay of inflammatory pain. However, CM-304 [17.5 (12.7–25.2) mg/kg, i.p.) and AZ-66 [11.6 (8.29–15.6) mg/kg, i.p.) were less efficacious than morphine [3.87 (2.85–5.18) mg/kg, i.p.] in the 55°C warm-water tail-withdrawal assay. While AZ-66 exhibited modest sedative effects in a rotarod assay and conditioned place aversion, CM-304 did not produce significant effects in the place conditioning assay. Overall, these results demonstrate the S1R selective antagonist CM-304 produces antinociception and anti-allodynia with fewer liabilities than established therapeutics, supporting the use of S1R antagonists as potential treatments for chronic pain.

## Introduction

Chronic pain is the number one cause of adult disability in the United States. According to the National Institutes of Health, an estimated 20 million individuals suffer from some form of peripheral neuropathy (NINDS, 2018). Current existing primary treatments for managing chronic pain include anticonvulsants (i.e., gabapentin, pregabalin), followed by secondary treatments including tricyclic antidepressants (e.g., amitriptyline, desipramine) and mu opioid receptor (MOR)-selective agonists (e.g., morphine), but the liabilities of these treatments greatly offset their therapeutic benefits (Yaksh and Wallace, 2011). These agents all cause drowsiness and impair locomotor ability, posing a significant risk when operating machinery and increasing the risk of falling, which in the elderly has been linked to increased mortality risk (Calandre et al., 2016; Mangram et al., 2016). More concerning is the potential for MOR agonists to demonstrate tolerance as well as opioid-induced hyperalgesia (DeLeo et al., 2004), and produce constipation, respiratory depression, substance abuse and addiction (Rosenblum et al., 2008). Overall, there remains a clear need for new, safer non-opioid options to treat chronic pain.

Sigma receptor antagonists are emerging as potential therapeutics and adjuvants to treat nerve injury, neuroinflammation, and the modulation of nociception (Vidal-Torres et al., 2013; Davis, 2015). Although once thought to be a member of the opioid family (Martin et al., 1976) or a binding site on N-Methyl-D-aspartate (NMDA) receptors (Wong et al., 1988), subsequent cloning of the sigma-1 (S1R; Hanner et al., 1996) and sigma-2 receptors (S2R; Alon et al., 2017) is leading to a more defined role of this system in biological systems. In particular, S1R are thought to play an active modulatory role in pain signaling, both centrally and peripherally (Kim et al., 2008; Roh et al., 2011). Sigma-1 receptors (S1Rs) are intracellular chaperone proteins (Walker et al., 1990) that modulate both central sensitization of pain (Gris et al., 2016) as well as oxidative stress (Pal et al., 2012). S1Rs were reported to be upregulated at the site of partial sciatic nerve ligation (Shen et al., 2017b), and pharmacological antagonism with the early selective sigma receptor antagonist E52862 reduced neuropathic nociception and spinal sensitization (de la Puente et al., 2009; Romero et al., 2012), and has been found effective in treating neurogenic pain (Wunsch, 2012). Notably, existing commonly used antagonists have limited specificity between the sigma receptors (BD1067) and sometimes significant affinity for other targets (notably Haloperidol; Matsumoto and Pouw, 2000; Matsumoto et al., 2001). However, the recent translational validation of E52862 as an efficacious treatment for oxaliplatin-induced neuropathy in a phase II clinical trial (Bruna et al., 2018) has reinvigorated interest in the development of newer, selective sigma receptor antagonists.

**Figure 1:**
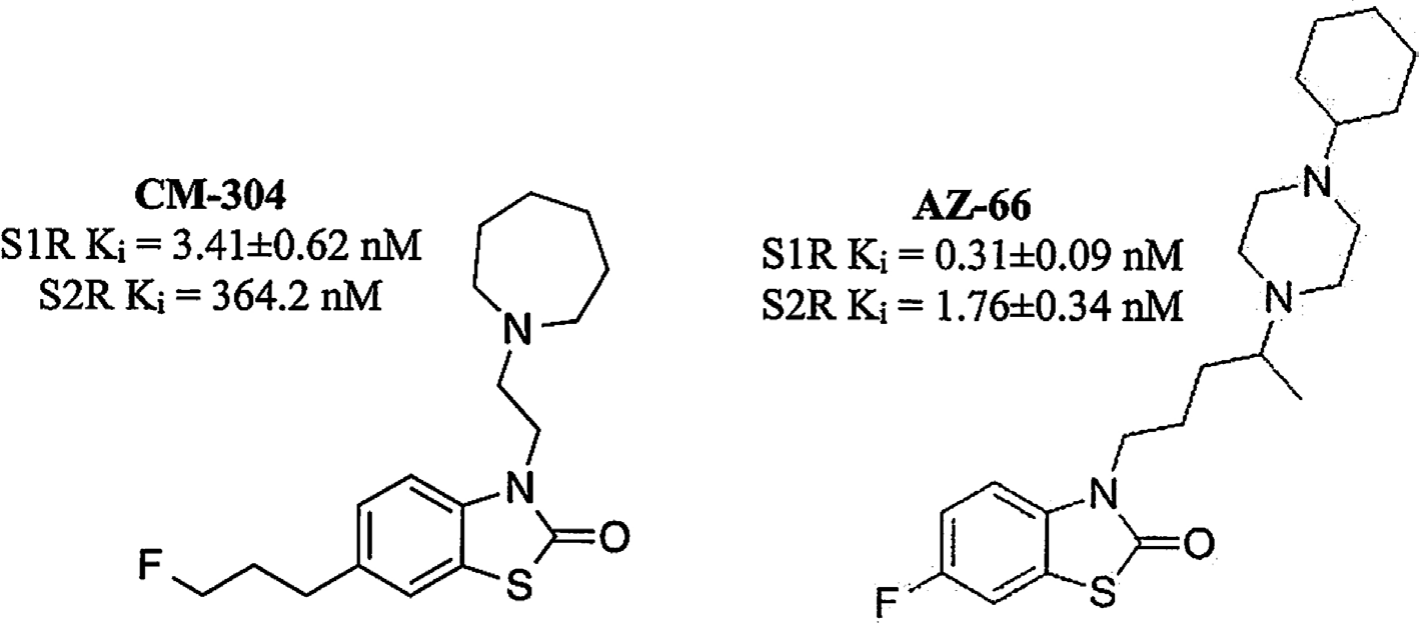
Structures of **CM-304** and **AZ-66**.

The recent characterization of CM-304 (**Figure 1**) found it to be a selective S1R antagonist, with >100-fold selectivity over S2R, and >10,000-fold selectivity over 59 other targets, including opioid and 5-HT receptors (James et al., 2012; James et al., 2014). The autoradiographic labeling of FTC146, the radiolabeled analog of CM-304, was abolished in S1R knock out mice, further demonstrating the S1R selectivity of this antagonist (Shen et al., 2015). While readily penetrating the CNS, CM-304 possesses a short *in vivo* half-life (115 min) and modest clearance (Cl = 33 ml/min/kg) (Avery et al., 2017). Seeking to improve the pharmacokinetics of this selective S1R antagonist, the analog AZ-66 was developed and shown to be a longer-lasting antagonist that possesses high affinity for both the S1R and S2R (Seminerio et al., 2012; Jamalapuram et al., 2013; Avery et al., 2017; **Figure 1**).

We hypothesized that the S1R selective antagonist CM-304 and non-selective S1R/S2R antagonist AZ-66 would produce significant anti-allodynic and antinociceptive effects in mouse models of chronic, induced pain with fewer liabilities of use as displayed by established analgesic agents. Activity of the two antagonists was examined in mouse assays of thermal (tail-flick), chemical (acetic acid), and induced inflammatory pain (formalin), as well as the chronic nerve constriction injury (CCI) and cisplatin-induced neuropathy (CISN) models of neuropathic pain and allodynia. Furthermore, C57BL/6J mice administered CM-304 and AZ-66 were examined for respiratory, locomotor, and sedative effects using the Comprehensive Lab Animal Monitoring System (CLAMS) and rotarod assay, and possible rewarding or aversive effects with the conditioned place preference (CPP) assay.

## Methods

### Subjects

Adult male C57BL/6J (The Jackson Laboratory, Bar Harbor, ME, USA) and CD-1 (Charles River Laboratories, Wilmington, MA, USA) mice were housed five to a cage, and tested at 8–12 weeks of age. C57BL/6J mice are established subjects in antinociceptive (Mogil et al., 1996; Wilson et al., 2003) respiratory and locomotor (Reilley et al., 2010) and place-conditioning assays (Brabant et al., 2005; Orsini et al., 2005). Analgesic effects were further confirmed in CD-1 mice, a strain also well validated for antinociceptive (Mogil et al., 2005) and thermal and mechanical anti-allodynic testing (LaCroix-Fralish et al., 2005; Feehan et al., 2017). Animal studies are reported in compliance with the ARRIVE guidelines (Kilkenny et al., 2010; McGrath and Lilley, 2015). Final sample sizes (i.e., a fixed number of animals for a particular test) were not predetermined by a statistical method, and animals were assigned to groups randomly. Drug treatment experiments were conducted in a blinded fashion. No animals were excluded from statistical analysis.

Mice were housed in a temperature and humidity controlled room at the University of Florida (Gainesville, Florida, USA) vivarium on a 12:12-h light/dark cycle with free access to food and water except during experimental sessions. All procedures were preapproved and conducted in accordance with the Institutional Animal Care and Use Committee at the University of Florida as specified by the 2011 NIH *Guide for the Care and Use of Laboratory Animals*. Upon the completion of testing, all mice were euthanized by inhalation of carbon dioxide, followed by cervical dislocation as a secondary measure, as recommended by the American Veterinary Medical Association.

### Materials, Drug Preparation, and Administration

The sigma receptor antagonists CM-304 and AZ-66 were synthesized and isolated as hydrochloride salts as described previously (McCurdy et al., 2014). All drugs and chemicals otherwise used were purchased from Sigma-Aldrich (St. Louis, MO, USA). For experiments, sterile isotonic saline (0.9%) was used to dissolve drugs to desired concentrations for testing (morphine, U50,488, E52862, AZ-66, and CM-304, 0.3–4.5 mg/ml). Gabapentin was dissolved in 5% Dimethyl sulfoxide (DMSO)/isotonic saline to 5.0 mg/ml concentration. All drugs were administered intraperitoneally (i.p.) in a volume of 250 µl per 25 g body weight.

### Behavioral Assays

#### Chronic Constriction Injury

Chronic constriction injuries (CCIs) were made to isoflurane (2.5%) anesthetized CD-1 mice (Hoot et al., 2010). This manipulation induces hyperalgesia and mechanical allodynia within 7 days, modeling neuropathic pain (Bennett and Xie, 1988; Pitcher et al., 1999; Cheng et al., 2002; Xu et al., 2004). Briefly, mice were anesthetized and an incision made along the surface of the biceps femoris (Hoot et al., 2010). Blunt forceps were inserted into the muscle belly to split the muscle and expose the right sciatic nerve. The tips of the forceps were passed under the sciatic nerve to pass two 5–0 chromic gut sutures (Ethicon, Cornelia, GA) under the nerve, 1 mm apart. The sutures were tied loosely around the nerve and knotted twice to prevent slippage, and skin was closed with 2–3 ligatures of 6–0 nylon. Mice were allowed to recover for 7 days prior to the initiation of testing. Mice so injured were confirmed hypersensitive to tactile stimulation with a series of von Frey hairs prior to testing, typically removing the ipsilateral paw from contact with just ∼20% of the baseline force required. Animals in neuropathic pain are hypersensitive to tactile stimulation (allodynia), and respond to lower pressure. Allodynic mice were then administered (i.p.) either vehicle (5% DMSO), morphine (10 mg/kg, i.p.), E52862 (30 mg/kg, i.p.), gabapentin (50 mg/kg, i.p.), CM-304 or AZ-66 (10–45 mg/kg, i.p., each). Every 20 min for 80 min, the threshold for tactile allodynia was measured using a series of calibrated von Frey filaments possessing a bending force from 0.4 to 6 g until the threshold that induced paw withdrawal was found as a measure of nocifension (Bennett and Xie, 1988; Pitcher et al., 1999; Cheng et al., 2002; Xu et al., 2004). The filaments were applied by ascending strength, and threshold was defined as two withdrawals per trial of the same filament strength. Responsiveness to von Frey fibers is indicative of mechanical allodynia as uninjured mice do not respond with paw withdrawal at these strengths. Data are expressed as percent of baseline paw withdrawal thresholds following stimulation of the ipsilateral hind paw with von Frey filaments. This was utilized to account for innate variability between mice. % antiallodynia = 100 × ([mean paw withdrawal force {g} in control group − paw withdrawal force {g} of each mouse]/mean paw withdrawal force [g] in control group).

#### Cisplatin-Induced Neuropathy Assay

The effectiveness of the sigma-receptor antagonists or control compounds against a chemically induced neuropathy, produced by treatment with cisplatin (2.3 mg/kg, i.p.) on alternating days with lactated Ringer’s solution on intervening days over a 9-day period, was determined (Zhou et al., 2016). Drug efficacy screening was conducted on day 10 to minimize the potential effect that repeated testing may have on endpoints. Anti-allodynic effects against mechanical allodynia were determined with measurements using a series of calibrated von Frey monofilaments, as described above in the CCI assay.

#### Acetic Acid Stretching Assay

Antinociceptive efficacy against visceral, chemical pain using the acetic acid stretching assay was assessed with C57BL/6J mice as described previously (Bidlack et al., 2002; Eans et al., 2015). Twenty-five minutes after receiving a single dose of test drug, an i.p. injection of 0.9% acetic acid (0.25 ml per 25 g body wt.) was administered to each mouse. After 5 min, the number of stretches displayed by each mouse was counted for an additional 15 min. Antinociception for each tested mouse was calculated by comparing the test group to a control group in which mice were treated with the appropriate vehicle (i.p.) using the formula:

% antinociception=([{average stretches in the vehicle group}−{number of stretches in each test mouse}]/[average stretches in vehicle group])

#### Formalin Assay

Additional testing of antinociceptive potency against inflammatory pain was performed using the formalin assay in C57BL/6J mice as previously described (Cheng et al., 2002). Following a 30-min pretreatment of a single dose of vehicle control or test drug (i.p.), an intraplantar (i.pl.) injection of 5% formalin (2.5 μg in 15 µl) was administered into the right hind paw. Paw-licking duration was recorded in 5-min intervals for 60 min following injection. The last 55 min was used to determine response to an inflammatory stimulus. Data were analyzed as area under the curve (AUC) representing summed time mice spent licking their inflamed hind paw.

#### Tail-Withdrawal Assay

The 55°C warm-water tail-withdrawal assay was conducted in C57BL/6J mice as a measure of acute thermal antinociception as described previously (Reilley et al., 2010). Briefly, each mouse was tested for baseline tail-withdrawal latency prior to drug administration. Following drug administration (i.p.), the latency for each mouse to withdraw the tail was measured every 10 min until latency returned to the baseline value. A maximum response time of 15 s was utilized to prevent tissue damage. If the mouse failed to display a tail-withdrawal response within 15 s, the tail was removed from the water and the animal was assigned a maximal antinociceptive score of 100%. Data are reported as percent antinociception, calculated by the equation: % antinociception = 100 × ([test latency−baseline latency]/[15−baseline latency]). This was utilized to account for innate variability between mice.

#### CLAMS Measurement of Respiration Rate and Spontaneous Locomotor Testing

Respiration rates and spontaneous ambulation rates were monitored using the automated, computer-controlled Comprehensive Lab Animal Monitoring System (CLAMS) (Columbus Instruments, Columbus, OH) as described previously (Reilley et al., 2010). Freely moving mice were habituated in closed, sealed individual apparatus cages (23.5 cm × 11/5 cm × 13 cm) for 60 min before testing. To start testing, mice were administered (i.p.) drug or vehicle and 5 min later confined to the CLAMS testing cages for 120 min. Using a pressure transducer built into the sealed CLAMS cage, the respiration rate (breaths/min) of each occupant mouse was measured. Infrared beams located in the floor measured locomotion as ambulations, from the number of sequential breaks of adjacent beams. Data are expressed as percent of vehicle control response.

#### Rotarod Assay to Assess Motor Coordination

Possible sedative effects of vehicle, morphine, U50,488, CM-304 or AZ-66 were assessed by rotarod performance, as described previously (Eans et al., 2015). Following seven habituation trials (the last utilized as a baseline measure of rotarod performance), mice were administered (i.p.) test agent: vehicle (5% DMSO/95% saline; 12 mice), morphine or U50,488 (10 mg/kg, i.p. each, eight mice each), or CM-304 or AZ-66 (45 mg/kg, i.p. each; 10 mice each) and assessed after 10 min in accelerated speed trials (180 s max latency at 0−20 rpm) over a 60-min period, measuring time to fall (in seconds). To normalize for each mouse’s individual performance, data are expressed as the average of the percent change from baseline performance for each mouse. Decreased latencies to fall in the rotarod test indicate impaired motor performance.

#### Conditioned Place Preference

An automated, balanced three-compartment place conditioning apparatus (San Diego Instruments, San Diego, CA) and 2-day counterbalanced conditioning design was used (similar to Eans et al., 2013). Prior to place conditioning, mice were allowed free access to all three chambers of the apparatus for 30 min to determine initial outer chamber preference. Time spent in each chamber was recorded. Prior to place conditioning, the 98 animals tested did not demonstrate significant differences in their time spent exploring the left (552.8 ± 12.5 s) versus right (590.8 ± 12.9 s) compartments (P = 0.09; Student’s t-test). Each day mice were administered assay vehicle (0.9% saline, i.p.) and consistently confined in a randomly assigned outer compartment (i.e., half of each group in the right chamber, half in the left chamber). Four hours later, C57BL/6J mice were administered compound and confined to the opposite compartment for 40 min of place conditioning in the appropriate outer compartment. All place conditioning was repeated for a second day, and final place preference was determined 24 h later. Data are plotted as the difference in time spent in the eventual conditioning-drug paired and vehicle-paired compartments. By convention, a positive value reflects a conditioned preference and a negative value conditioned aversion for the drug-paired side. Results compared the pre-conditioning responses and post-conditioning responses between sets.

#### Control Testing

To validate assay function, comparison control experiments for each assay (either negative controls with vehicle, or positive controls with agents such as morphine or gabapentin) were performed in small cohorts alongside testing of novel compounds throughout the study.

### Statistical Analysis

The data and statistical analysis comply with the recommendations on experimental design and analysis in pharmacology (Curtis et al., 2015). All data are presented as mean ± SEM, with a significance set at P < 0.05, denoted by the asterisk (*). All data were statistically evaluated with Prism 7.0 software (GraphPad Software, La Jolla, California, USA). All statistical analysis were examined for normality and equal variance using GraphPad. All data demonstrated normality and equal variance, justifying parametric analysis. Dose response lines were analyzed by linear or nonlinear regression modeling and ED_50_ values (dose yielding 50% effect) along with 95% confidence limits using each individual data points. CLAMS data are reported as the % of matching vehicle control responses. The rotarod data are expressed as the % change from baseline performance, a standard normalization that compensates for each individual animal’s baseline response. CPP data are reported as the difference in time spent in the drug- and vehicle-paired compartments between pre-conditioning and post-conditioning responses. Significant differences in behavioral data were analyzed by ANOVA (one-way or two-way repeated measures), with significant results further analyzed with Dunnett’s, Sidak, or Tukey’s Honestly significant difference (HSD) *post hoc* tests as appropriate for significant pairwise comparisons within and between groups.

## Results

### Sigma Receptor Antagonists Dose-Dependently Alleviate Multiple Modalities of Induced Nociception

**Figure 2:**
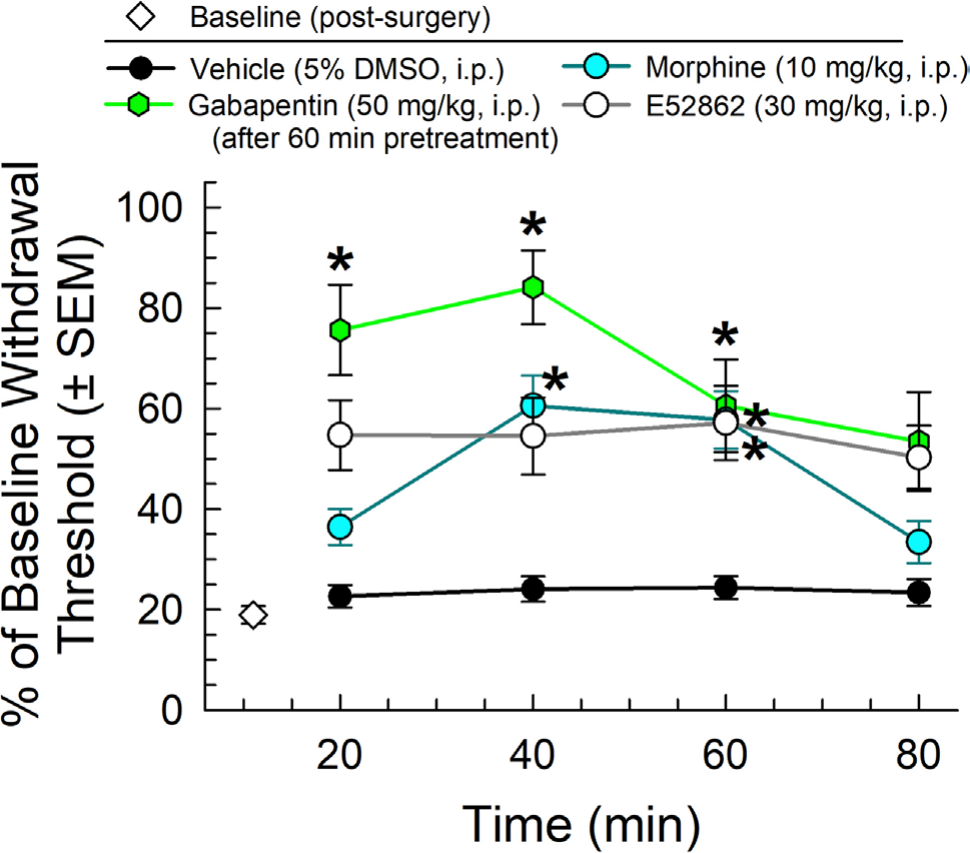
Dose- and time-dependent antiallodynic activity of morphine (blue circles), gabapentin (green hexagons), or the sigma-receptor antagonist E52862 after i.p. administration in the mouse chronic constriction injury (CCI) assay. * = significantly greater than vehicle effect (black circles) at matching time points, p < 0.05; two-way ANOVA w/Tukey’s *post hoc* test. N = 10–12 mice/point.

We first completed the characterization of a set of established control analgesics in the mouse CCI assay of neuropathic pain. Following administration through the intraperitoneal (i.p.) route and using von Frey filaments to measure mechanical allodynia, the mu-opioid receptor agonist, morphine (10 mg/kg), the sigma-receptor antagonist E52862 (30 mg/kg), and the established treatment for neuropathic pain, gabapentin (50 mg/kg, given 60 min prior to testing), all significantly attenuated the reduced paw withdrawal threshold caused by CCI (factor *treatment*: F_(3,151)_ = 4.01, p = 0.009 and factor *time*: F_(3,151)_ = 31.7, p < 0.0001; two-way ANOVA and Tukey’s multiple comparison *post hoc* test; **Figure 2**). These results were consistent with established observations 1) that gabapentin produces antiallodynia useful in the treatment of neuropathic pain, 2) that sigma receptor antagonists as represented by E52862 may also produce antiallodynia, and 3) that morphine, while somewhat efficacious, is less effective than gabapentin (p < 0.05, 20 and 40 min time points).

**Figure 3:**
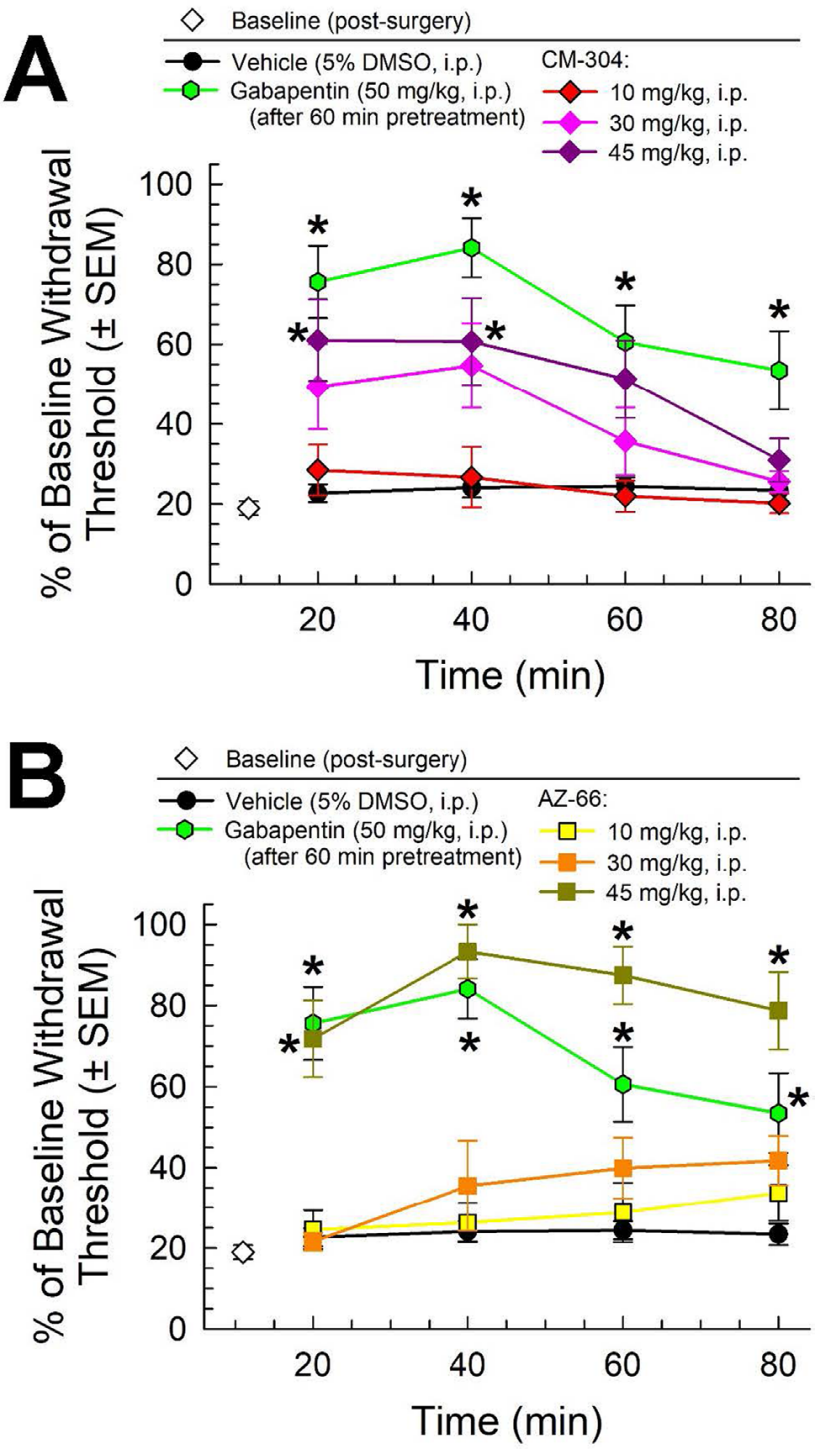
Dose- and time-dependent antiallodynic activity of **(A)** CM-304 (diamonds) and **(B)** AZ-66 (squares) in the mouse chronic constriction injury (CCI) assay. Gabapentin (green hexagons, 60-min pretreatment) is included as a positive control; vehicle (5% DMSO; black circles) is included as a negative control. * = significantly greater than vehicle effect, p < 0.05; two-way ANOVA w/Tukey *post hoc* test. N = 8–10 mice treated with a single dose of sigma receptor antagonist and 11 mice for gabapentin.

Following i.p. administration, both CM-304 and AZ-66 demonstrated anti-allodynic effects in the CCI assay. CM-304 (**Figure 3A**) produced significant relief of CCI-induced allodynia in a dose- and time-dependent manner (factor *treatment*: F_(4,171)_ = 26.11, p < 0.0001 and factor *time*: F_(3,171)_ = 6.71, p = 0.0003; two-way ANOVA and Tukey’s *post hoc* test). These effects were short-lasting (less than 60 min) even at the highest dose, consistent with the known rapid metabolism of this ligand. In contrast, the antiallodynic efficacy of AZ-66 (**Figure 3B**) was significant only at the higher dose tested (45 mg/kg; factor *treatment*: F_(4,171)_ = 51.4, p < 0.0001), but it was also significantly elevated above the effects of gabapentin for an extended period (p = 0.04; 60 min time point, Student’s t-test). The completion of this testing confirms the anti-allodynic activity of both S1R and mixed affinity S1R/S2R antagonists against neuropathic pain.

**Figure 4:**
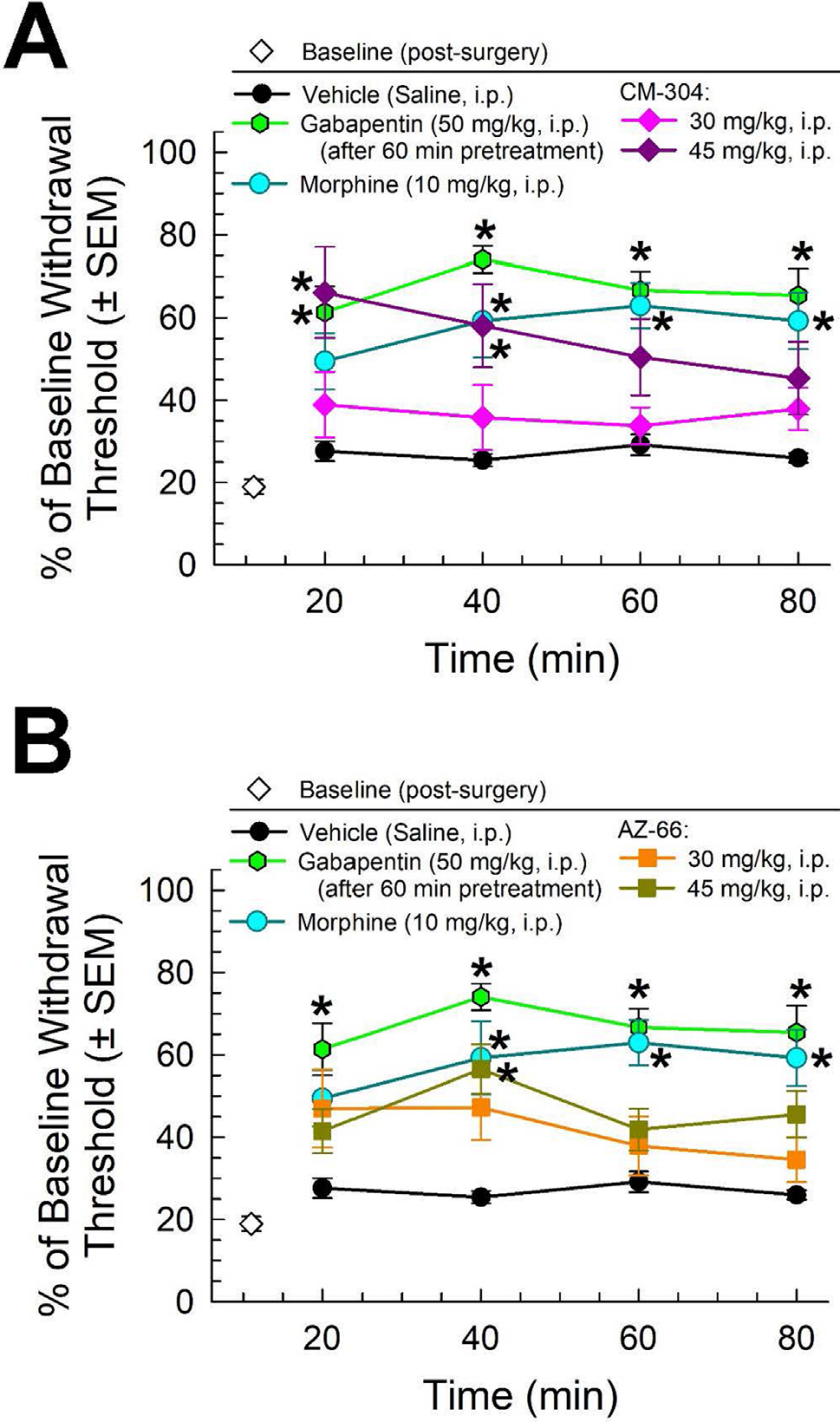
Dose- and time-dependent antiallodynic activity of **(A)** CM-304 (diamonds) and **(B)** AZ-66 (squares) in the mouse chemotherapy-induced neuropathy (CISN) assay. Morphine (blue circles) and gabapentin (green hexagons, 60-min pretreatment) are included as positive controls; vehicle (saline; black circles) is included as a negative control. * = significantly greater than vehicle effect, p < 0.05; two-way ANOVA w/Tukey’s *post hoc* test. N = 9–10 mice treated with a single dose of sigma receptor antagonists, six mice treated with morphine, nine mice treated with gabapentin, and eight mice treated with saline alone.

Similarly, morphine (10 mg/kg) and gabapentin (50 mg/kg, given 60 min prior to testing) all significantly attenuated the reduced paw withdrawal threshold caused by chronic exposure to CISN (factor *treatment*: F_(2,80)_ = 75.3, p < 0.001; two-way ANOVA and Tukey’s multiple comparison *post hoc* test; **Figure 4**). The sigma receptor antagonists demonstrated modest anti-allodynic effects in the CISN assay, with significant dose-dependent effects upon treatment with higher doses (45 mg/kg) of either CM-304 (factor *treatment*: F_(2,100)_ = 14.48, p < 0.0001; **Figure 4A**) or AZ-66 (factor *treatment*: F_(2,96)_ = 10.58, p < 0.0001; **Figure 4B**).

**Figure 5:**
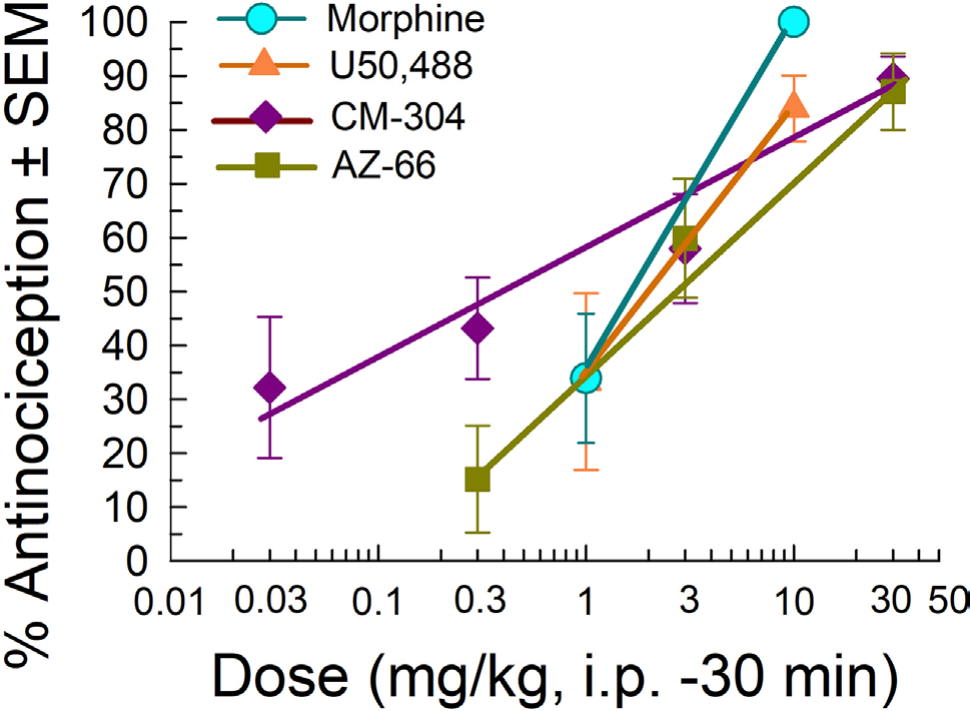
Dose-dependent antinociception of sigma-receptor antagonists CM-304 and AZ-66 following i.p. administration in the mouse acetic-acid writhing assay. Opioid agonists morphine and U50,488 are shown as positive controls. All points represent average response ± SEM at peak effect, 30 min after admin in 8–14 mice.

We further evaluated the sigma receptor antagonists CM-304 and AZ-66 in the mouse acetic acid writhing test and formalin assay to evaluate visceral and inflammatory pain, respectively. On the basis of the activity in the neuropathic pain assays, we administered AZ-66 and CM-304 through the intraperitoneal route and examined antinociceptive efficacy *in vivo* in mouse models of visceral, chemical pain (the acetic-acid writhing assay; **Figure 5**). The sigma-receptor antagonists produced dose-dependent antinociception in the writhing assay, with ED_50_ values (and 95% confidence intervals) of 0.48 (0.09–1.82) mg/kg, i.p. (CM-304) and 2.31 (1.02–4.81) mg/kg, i.p. (AZ-66). These effects are comparable to the analgesia of the established opioid agonists morphine [1.75 (0.27–1.15) mg/kg, i.p.] and U50,488 [2.13 (0.04–49.8) mg/kg, i.p.] (**Figure 5**).

**Figure 6:**
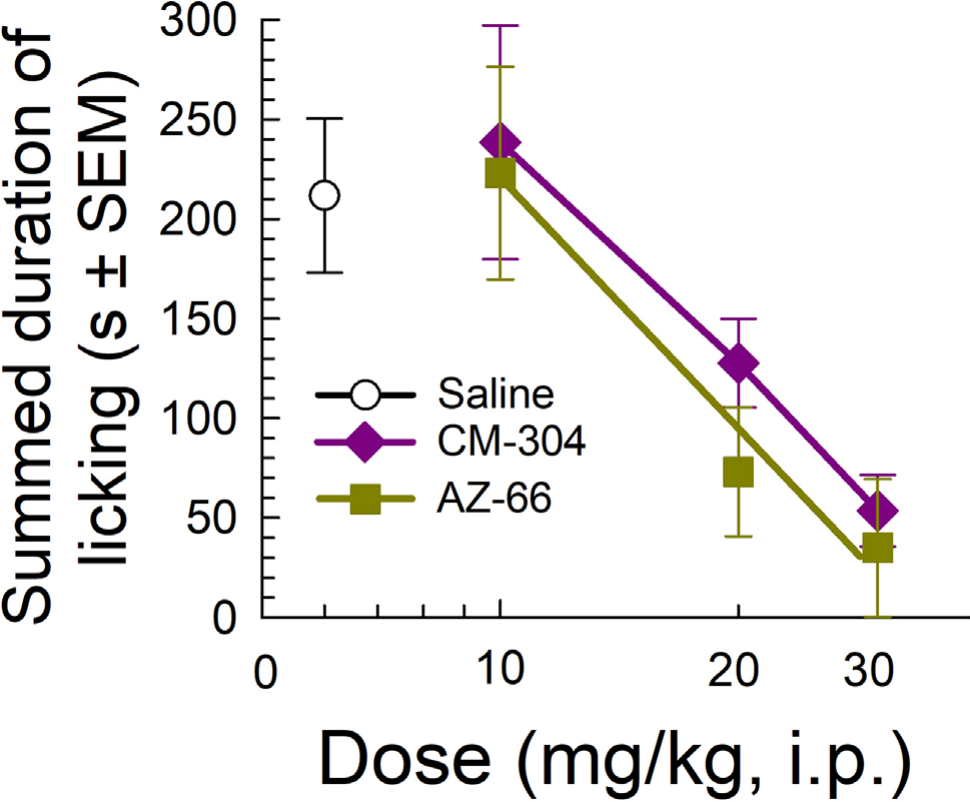
Dose-dependent antinociception of sigma-receptor antagonists CM-304 and AZ-66 following i.p. administration in the mouse formalin assay. Control mice treated with saline (0.9%, i.p.; n = 8). All points represent average response ± SEM administered to 8–10 mice.

Likewise, testing of the sigma-receptor antagonists in the mouse formalin assay showed significant dose-dependent analgesic efficacy against inflammatory pain, with both CM-304 and AZ-66 equally reducing the amount of time that animals spent licking the inflamed paw in a dose-dependent manner as compared to vehicle-treated mice (F_(3,33)_ = 4.93, p = 0.006 and F_(3,34)_ = 5.51; p = 0.003, respectively; one-way ANOVA w/Dunnett’s *post hoc* test; **Figure 6**).

**Figure 7:**
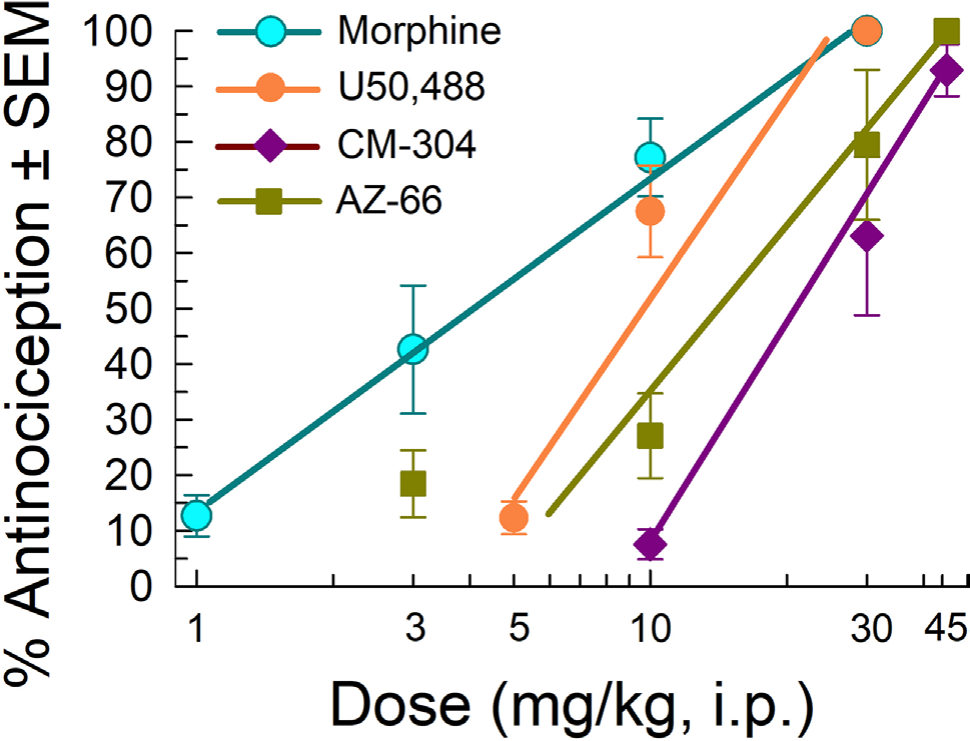
Testing efficacy against acute thermal nociception in the 55°C warm-water tail-withdrawal test. Opioid agonists morphine and U50,488 are shown as positive controls. Morphine and U50,488 points represent average response ± SEM at peak effect, 30 min after admin in eight or 16 mice. All points with CM-304 and AZ-66 represent average response ± SEM at peak effect, 20 min after admin in eight mice/dose tested.

We next evaluated the antinociceptive abilities of CM-304 and AZ-66 against a thermal nociceptive stimulus in the mouse 55°C warm-water tail-withdrawal assay. The MOR agonist morphine and KOR agonist U50,488 produced dose-dependent antinociception with ED_50_ and 95% C.I. values of 3.87 (2.85–5.18) and 8.11 (6.19–9.94) mg/kg, i.p., respectively (**Figure 7**). In contrast, AZ-66 exhibited antinociception with an ED_50_ value of 11.6 (8.29–15.6) mg/kg, i.p., while the selective S1R antagonist CM-304 produced antinociception with an ED_50_ value of 17.5 (12.7–25.2) mg/kg, i.p., significantly less efficacious than morphine (F_(2,145)_ = 17.3; p < 0.0001; nonlinear regression modeling).

### Evaluation of CM-304 and AZ-66 *In Vivo* for Potential Clinical Liabilities

**Figure 8:**
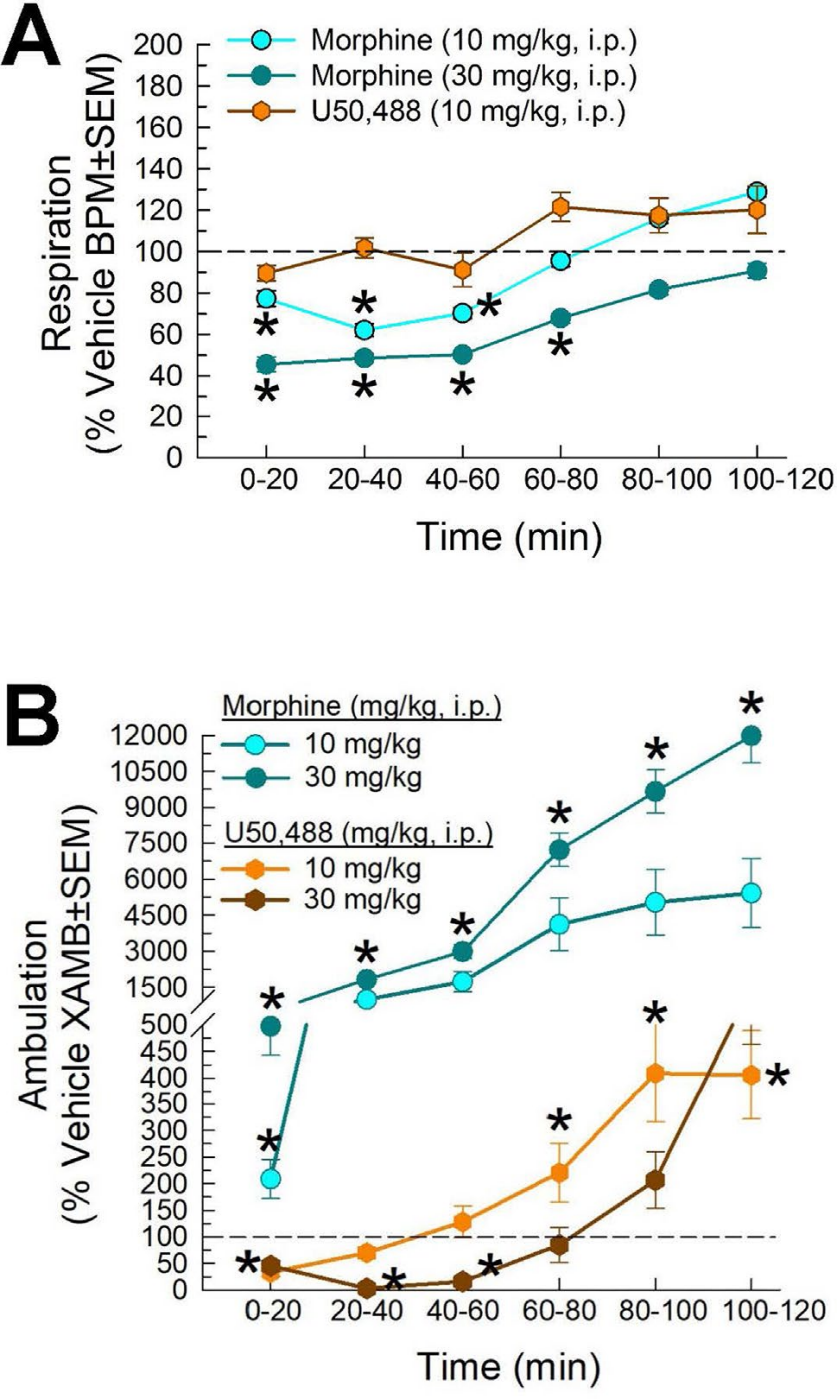
Dose- and time-dependent **(A)** respiratory depression and **(B)** spontaneous locomotor effects of morphine (cyan circles) or U50,488 (orange hexagons) evaluated in the CLAMS assay with C57BL6/J mice. * = significantly greater than vehicle effect (dashed line), p < 0.05; two-way ANOVA w/Dunnett’s *post hoc* test. N = 16–20 mice/group.

Following administration through the intraperitoneal route, the mu-opioid receptor agonist, morphine (10 and 30 mg/kg), and the kappa-opioid-receptor agonist U50,488 [10 mg/kg; and 30 mg/kg not shown)] showed different results on respiration rate in the CLAMS. Compared to vehicle, morphine significantly reduced respiration rate (factor *treatment* × *time*: F_(20,420)_ = 2.05, p = 0.005; two-way ANOVA and Dunnett’s multiple comparison *post hoc* test; **Figure 8A**). In contrast, U50,488 did not significantly reduce respiration at any time point (and, in fact, showed a trend toward increased respiration). Likewise, significant differences of drug-induced locomotion were observed (factor *treatment* × *time*: F_(20,429)_ = 36.4, p < 0.0001; two-way ANOVA and Dunnett’s *post hoc* test), with morphine producing dose-dependent increased locomotor activity, while U50,488 suppressed spontaneous locomotion (**Figure 8B**). Overall, these results were consistent with established observations that mu-opioid agonists suppress respiration while producing psychostimulatory effects, key liabilities in clinical use.

**Figure 9:**
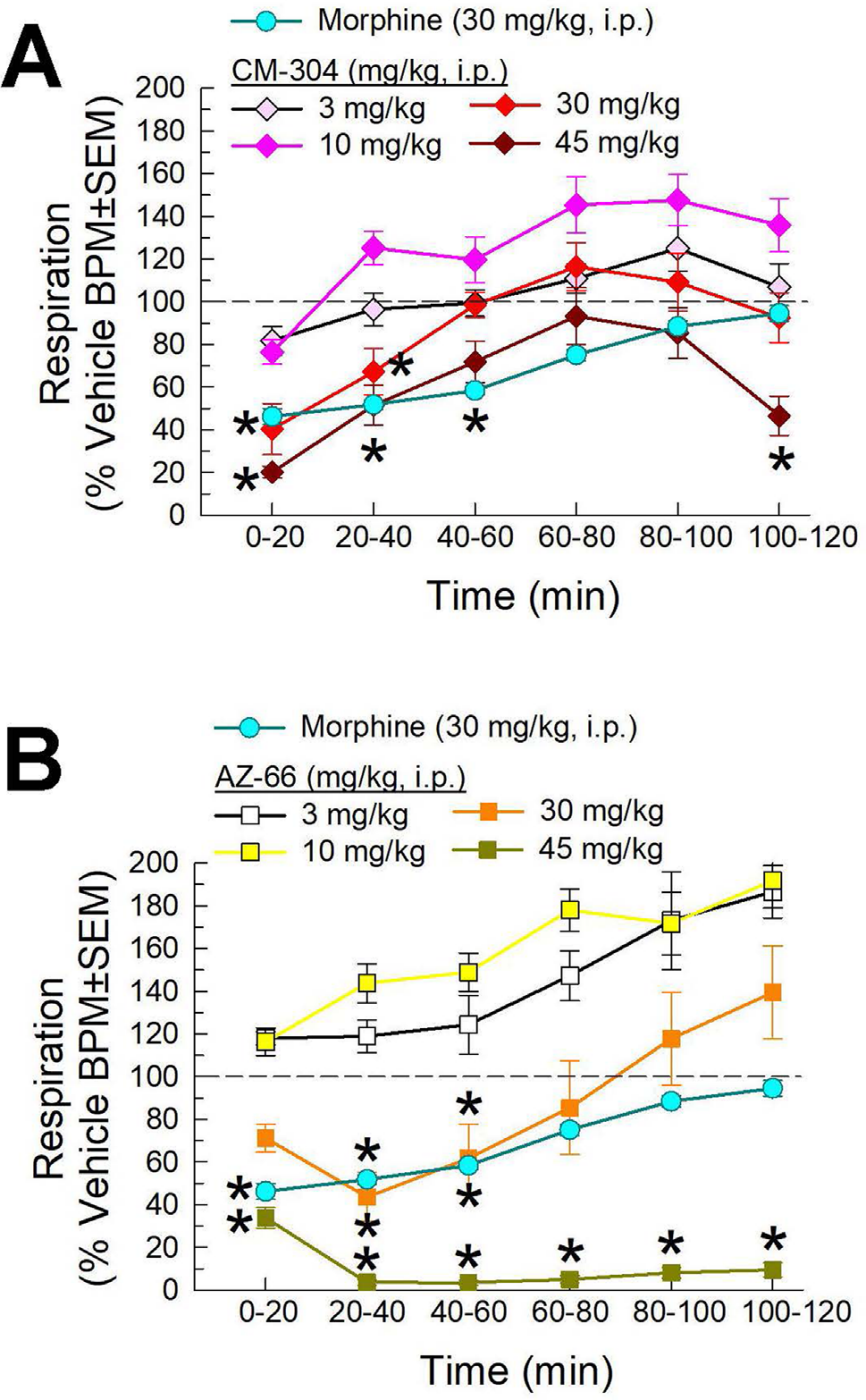
Dose- and time-dependent respiratory effects of i.p. administration of **(A)** CM-304 or **(B)** AZ-66 evaluated in the CLAMS assay with C57BL6/J mice. Morphine (30 mg/kg, i.p.) included as a positive control. * = significantly different from vehicle effect (dashed line), p < 0.05; two-way ANOVA w/Dunnett’s *post hoc* test. N = 10–20 mice/group.

We then completed the characterization of the respiratory effects of the lead compounds CM-304 and AZ-66. Following i.p. administration, both compounds demonstrated dose-dependent reductions in respiration in this assay. CM-304 (**Figure 9A**) and AZ-66 (**Figure 9B**) produced significant dose- and time-dependent respiratory depression (*treatment* × *time*: F_(25,504)_ = 2.31, p = 0.0004 and F_(25,450)_ = 2.81, p < 0.0001, respectively; two-way ANOVA and Dunnett’s multiple comparison *post hoc* test). Notably, these effects were more pronounced with AZ-66, with significant respiratory depression extending 2 h after administration of the 45 mg/kg, i.p. dose (**Figure 9B**).

**Figure 10:**
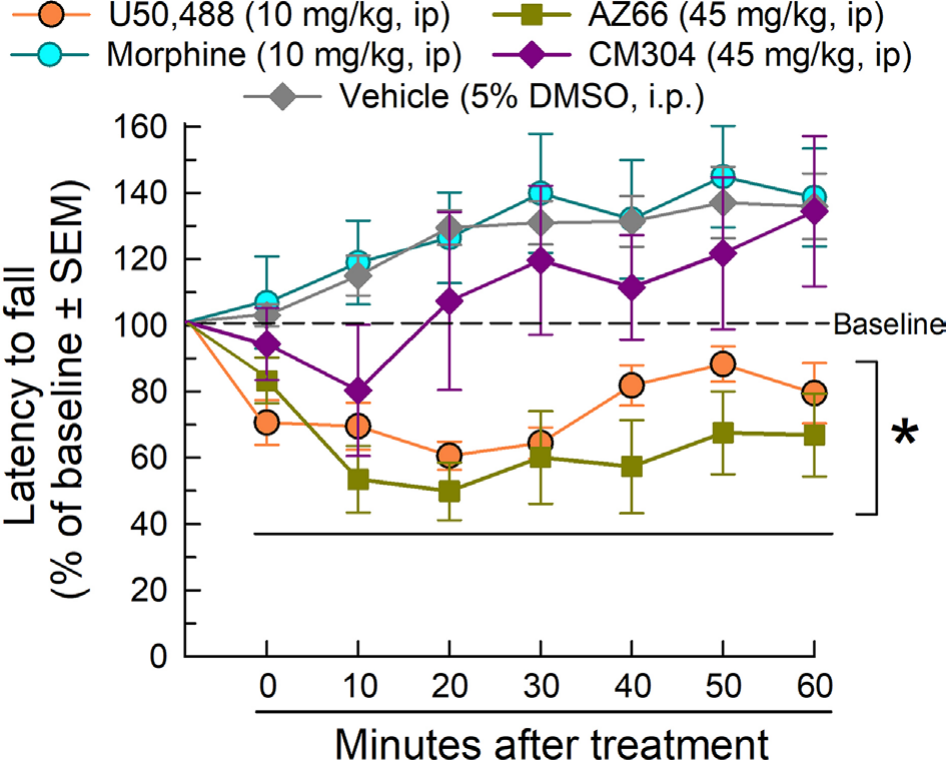
Dose- and time-dependent effects of CM-304 (purple diamonds) or AZ-66 (blue triangles) after a 45 mg/kg i.p. administration in the mouse rotarod assay. U50,488 (orange circles, 10 mg/kg, i.p.) is added as a positive control. * = significantly different than either vehicle effect (gray diamonds), p < 0.05; two-way ANOVA w/Dunnett’s *post hoc* test; N = 8–12 mice/treatment.

The rotarod assay is a measure of coordinated locomotor activity and sedation, measuring evoked locomotion that eliminates the potential complication of natural sleep during testing. AZ-66 and CM-304 were evaluated at the 45 mg/kg, i.p. dose that reduced respiration, yet proved to be effective in the neuropathic pain assays. In rotarod testing (**Figure 10**), morphine was without effect, but U50,488 significantly impaired locomotion as compared to vehicle (factor *treatment*: F_(4,301)_ = 36.5, p < 0.0001 and factor *time*: F_(6,301)_ = 2.44, p = 0.03; two-way ANOVA and Dunnett’s *post hoc* test). Whereas AZ-66 significantly impaired evoked locomotion over time, CM-304 did not significantly impair locomotion at any time tested. Confirming these findings, similar results were observed on ambulations measured in the CLAMS assay, presented as % vehicle effect. CM-304 was found to significantly (if modestly) increase ambulations in the second hour of testing (factor *treatment*: F_(4,354)_ = 10.2, p < 0.0001; two-way ANOVA and Dunnett’s *post hoc* test; **Supplemental Figure 1A**). Notably, although CM-304 did initially reduce raw ambulations in a dose-dependent manner (factor *treatment* × *time*: F_(20,345)_ = 5.72, p < 0.0001; two-way ANOVA and Dunnett’s *post hoc* test at 0–20 min; **Supplemental Figure 2B**), this effect was not significant when normalized to the response of vehicle-treated mice (p = 0.54, 0.77 and 0.99 for the 45 mg/kg, i.p. dose response across time points in the first hour; Dunnett’s *post hoc* test). Otherwise, consistent with the rotarod results, AZ-66 consistently produced a significant dose-dependent general reduction in ambulatory activity (factor *treatment*: F_(4,432)_ = 5.28, p = 0.0004 and factor *time*: F_(5,432)_ = 10.6, p < 0.0001; two-way ANOVA and Dunnett’s *post hoc* test; **Supplemental Figure 1B**).

### Evaluation of CM-304 and AZ-66 With the Mouse Conditioned Place Preference Assay

**Figure 11:**
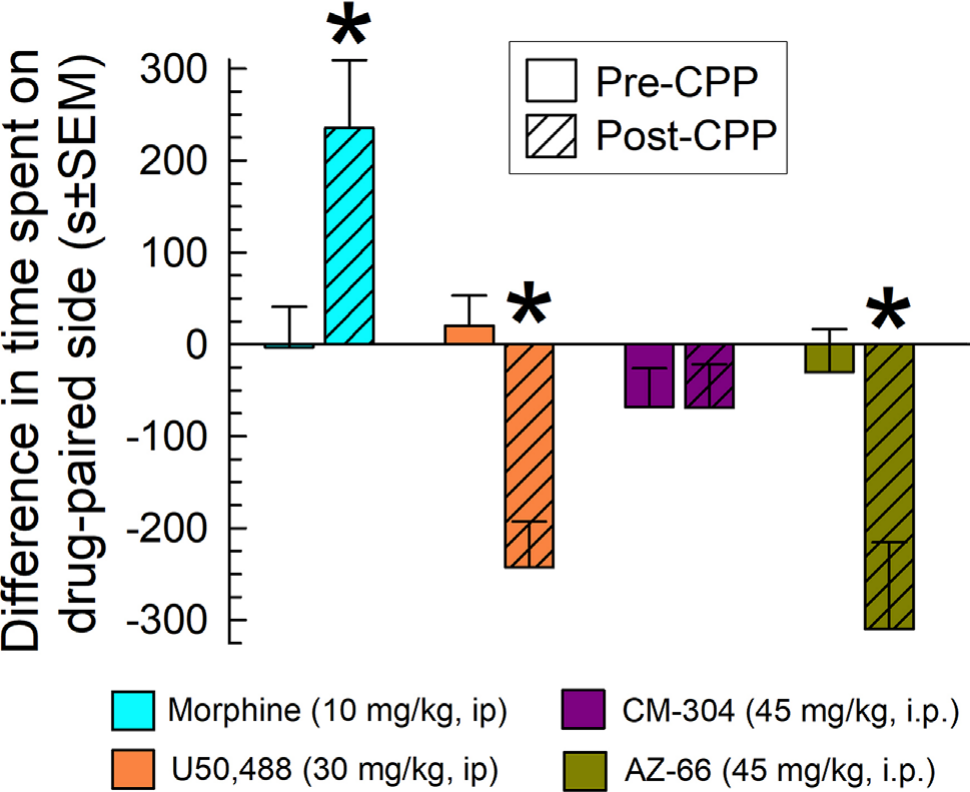
CM-304 (45 mg/kg/d, i.p.; N = 34) did not demonstrate place-conditioning preference (as did morphine, N = 17) or aversion, whereas AZ-66 (45 mg/kg/d, i.p., N = 19) demonstrated CPA similar to U50,488 (N = 28) in the mouse conditioned place preference assay. * = post-conditioning response (striped bars) significantly different from matching pre-CPP response (matching open bars), p < 0.05; two-way ANOVA w/Sidak’s *post hoc* test.

Mice were place conditioned for 40 min each of 2 days with morphine, the KOR-selective agonist U50,488, or the sigma-receptor antagonists CM-304 or AZ-66, using i.p. doses producing significant and consistent anti-allodynic effects (45 mg/kg). While morphine produced significant conditioned-place preference (CPP) and U50,488 produced conditioned-place aversion (CPA) (factor: *treatment* × *conditioning*: *F*_(3,194)_ = 8.79; *P* < 0.001; two-way ANOVA and Sidak’s multiple comparisons *post hoc* test), the S1R antagonist CM-304 produced place-conditioning responses similar to preconditioning responses (p = 0.99; **Figure 11**). In contrast, AZ-66 produced significant condition place aversion (p = 0.006; **Figure 11**).

## Discussion

Recent endeavors have demonstrated that the radiolabeled version of CM-304, [^18^F]-FTC-146, accumulates in the brain and periphery, is well tolerated, absorbed at acceptable doses in humans, and is able to accurately locate the site of nerve injury in rats (Hjornevik et al., 2017; Shen et al., 2017a; Shen et al., 2017b). As a viable PET agent, an early phase I clinical trial is investigating [^18^F]-FTC-146 distribution in patients suffering from complex regional pain syndrome (CRPS) and sciatica to determine how S1R expression is altered in chronic pain states in humans (ClinicalTrials.gov, 2016). While previous work examining AZ-66 and/or CM-304 have demonstrated their ability to prevent stimulant-induced neurotoxicity (Seminerio et al., 2012) and S1R agonist self-administration (Katz et al., 2016), their role as anti-nociceptive/allodynic agents have been evaluated for the first time here. The current data provide evidence that sigma receptor antagonists CM-304 and AZ-66 produced antinociceptive and anti-allodynic effects observed in behavioral assays of various modalities of inducible pain, while remaining less effective in a model of thermal reflexive pain. Although the non-selective S1R/S2R antagonist AZ-66 produced equivalent anti-allodynic, but longer-lasting effects in CCI compared to CM-304, it also produced mild locomotor impairment and conditioned place aversion (CPA). In contrast, the S1R selective antagonist CM-304 produced anti-allodynic effects without significant locomotor impairment or CPA. Further development of sigma receptor antagonists, in particular CM-304, may prove useful in providing relief to individuals suffering from poorly managed pain disorders like chronic pain.

CCI of the sciatic nerve and CISN are two commonly used rodent models of neuropathic pain, arising from sciatica and chemotherapeutic-associated pain respectively. An established “gold standard” for treating neuropathic pain, gabapentin, showed anti-allodynic efficacy at a dose (50 mg/kg i.p.) consistent with previous CCI and CISN studies (Ahn et al., 2009; Kinsey et al., 2009; Long et al., 2009; Ahmad et al., 2017), after a 1-h pretreatment to avoid confounding sedative effects. The present sigma receptor antagonists CM-304 and AZ-66 were as efficacious as the single dose of gabapentin in both models after immediate treatment with a dose of 45 mg/kg, i.p. Furthermore, we confirmed that the commercially available sigma-receptor antagonist E52862 is also efficacious in CCI at a similar dose (30 mg/kg). These data are consistent with the results of a recent phase II clinical study demonstrating E52862 efficacy in treating oxaliplatin-associated neuropathy in humans (Bruna et al., 2018). CCI and CISN both produce neuropathy but are thought to differ somewhat in underlying etiology associated with the development of allodynia. CCI is a focal injury of the sciatic nerve, and has been shown to upregulate sigma-1 receptor expression in the spinal cord, enhance central sensitization, and activate microglia in both the spinal cord and supraspinally throughout the brain (Roh et al., 2008; Barcelon et al., 2019). In comparison, the pathology of CISN may be more complex, with changes of morphology and molecular physiology of peripheral sensory nerves further associated with a neuronal inflammatory response that may impact both the peripheral and central nervous system in ways not yet fully understood to promote allodynia (Starobova and Vetter, 2017). Collectively, this underscores the limits of the current tests to ascertain where CM-304 or AZ-66 may be acting to prevent allodynia. For instance, the dorsal root ganglion (DRG), but not dorsal horn of spinal cord, has been implicated in the development of CISN-associated allodynia, but unlike CCI, CISN is not thought to activate spinal microglia (Zheng et al., 2011; Lessans et al., 2019), and the impact of chemotherapy on S1R expression in either the dorsal horn or DRG has yet to be elucidated. Future detailed investigations using S1R-CRE mice to evaluate the role of spinal, supraspinal, and peripheral sigma receptors in neuropathic pain might clarify this matter. Meanwhile, the current data support earlier reports demonstrating the therapeutic sensitivity of chemotherapy-induced neuropathy to S1R antagonism (Aley and Levine, 2002; Gris et al., 2016).

While CM-304 and AZ-66 demonstrated less efficacy in the 55°C warm-water tail withdrawal assay than morphine, this is consistent with previous literature (Vidal-Torres et al., 2013). Both sigma receptor antagonists were effective in treating inflammation-induced paw licking, supporting a broad therapeutic spectrum for the sigma receptor antagonists across the distinct etiologies contributing to neuropathic and inflammatory pain (Xu and Yaksh, 2011). The antinociception attributed to early sigma receptor antagonists was often found to be mediated by off-target effects such as opioid (Martin et al., 1976) or NMDA receptors (Wong et al., 1988). While affinity for other receptor targets associated with antinociception was not examined here, and thus directly discounted, it is notable that modern radiolabeled receptor competition binding assays have reported CM-304 to have both high affinity and selectivity for the S1R over 59 other receptor targets such as serotonin receptors (James et al., 2012; James et al., 2014), while AZ-66 had high affinity for both S1R and S2R (Seminerio et al., 2012), suggesting a role for these respective receptors in the current results. From what is known of nociception and sigma receptors, it is most feasible that CM-304 and AZ-66 exerted anti-allodynic and antinociceptive effects through antagonism of sigma-receptors. Inflammation and neuropathy exhibit similar increases in glutamatergic signaling and gliosis in the dorsal horn, immune cell invasion, and elevations of TNF-α in the DRG thought to sensitize nociceptive signaling. However, neuropathy but not inflammation is associated with an increase in voltage-dependent calcium channel subunit alpha-2/delta-1 in the DRG, a proposed site of action for gabapentinoids (Patel and Dickenson, 2016). Consistent with our current results, the administration of E52862 has been reported to reduce inflammatory allodynic responses induced by carrageenan and complete Freund’s adjuvant without altering carrageenan-induced paw edema, confirming that sigma antagonists modulate nociception without resolving the underlying pathology (Gris et al., 2014). Given that previous studies have indicated a higher density of sigma receptors in the DRG compared to the dorsal horn or supraspinal brain regions mediating nociception (specifically, the periaqueductal gray and basolateral amygdala), these results may suggest the dorsal root ganglia is a target of particular interest for sigma receptor involvement in the various and diverse modalities of pain (Sánchez-Fernández et al., 2014). Future anatomical and behavioral studies are expected to elucidate this concept.

Gabapentinoids like gabapentin have been reported to produce undesirable side effects including motor incoordination and respiratory depression that may lead to noncompliance or discontinuation, supporting the preclinical screening of novel therapeutics for these and other liabilities (Kaufman and Struck, 2011; Yaksh and Wallace, 2011; Evoy et al., 2017). Utilizing the CLAMS, both spontaneous locomotion and respiration were measured. Morphine, the prototypical MOR agonist, produced hyper-locomotion and decreases in respiration rate, while U50,488, a KOR-selective agonist, produced transient hypo-locomotion without altering respiration. CM-304 and AZ-66 significantly reduced respiration in a dose-dependent manner, although at a sub-therapeutic dose (10 mg/kg, i.p.) both compounds produced respiratory hyperventilation. It is not readily evident how CM-304 and AZ-66 induced respiratory depression, although the effects were more pronounced with AZ-66. To the best of our knowledge, this is the first time any sigma receptor antagonist has been evaluated for potential respiratory effects, a concern motivated by the current epidemic of opioid abuse. It is conceivable that sigma antagonists prevent S1R and/or S2R promotion of respiration. S1R RNA is heavily expressed in the medulla and less so in the hypothalamus (Lein et al., 2007). Both brain regions mediate arousal and sedation, suggesting possible modulation by sigma receptors in these behaviors. However, unlike the well-documented respiratory depression directly mediated by activation of mu opioid receptors in the brain’s respiratory network (Dahan et al., 2001), detailed investigation of sigma receptor mediation of breathing rate with plethysmography matched with electrophysiology of respiratory centers remains to be done. Alternatively, the sigma-receptor antagonists might indirectly affect respiration by decreasing locomotor activity. AZ-66 demonstrated disruption of coordinated locomotion in the rotarod assay, similar to U50,488, an agent known to produce motor incoordination and sedation (Zhang et al., 2015; Dunn et al., 2018). However, CM-304 was without significant inhibitory effects on locomotion, and in any case, the potential sedative effects of sigma-receptor antagonists are also not well understood. Further work is required to assess the effects of the sigma receptors (both sigma-1 and sigma-2) on respiration and locomotor activity, evaluating hypnotic vs. sedative effects. Future mapping studies of the distribution of S2Rs in brain and additional testing with new compounds showing selective antagonism for S2Rs may offer new insights into the role of sigma receptors in respiration, arousal, and sedation.

Substance abuse and addiction are additional concerns for the use of analgesics, given the epidemic of misused prescription opioids (Seth et al., 2018). To assess potential rewarding or aversive effects of CM-304 or AZ-66, we utilized the condition place preference/aversion assay (CPP/CPA). At supra-therapeutic dosing (45 mg/kg/day), CM-304 produced neither CPP or aversion, while AZ-66 unexpectedly produced conditioned place aversion similar to U50,488. The mechanism underlying the aversive effects of AZ-66 is not known. Kappa opioid receptor agonists produce dysphoria in humans (Pfeiffer et al., 1986) and conditioned place aversion in animals (Chefer et al., 2013), but AZ-66 does not demonstrate affinity for opioid receptors (Seminerio et al., 2012). It is conceivable that the present results suggest that S2R antagonists may produce aversion. Notably, previous work has demonstrated that neither CM-304 nor AZ-66 altered the reinstating effect of a priming dose of cocaine in rats demonstrating extinction after being trained to self-administer this psychostimulant (Katz et al., 2016). However, both sigma-receptor antagonists were able to block self-administration of the S1R agonists (+)-pentazocine or PRE-084 (Katz et al., 2016). Notably, these tests only examined reinstatement effects under extinction conditions, and with much lower individual doses (albeit through the same route) than tested here, but they highlight the burgeoning literature suggesting a modulating role for sigma receptors in reward and substance abuse. With the recent isolation of the S2R gene and anticipated transgenic animals, investigations into the specific contributions of S1R and S2R to reward or aversive states are expected to contribute new insights to this question. In the meantime, ongoing studies will evaluate whether selective S2R antagonists mimic AZ-66 conditioned place aversion, as well as the action of these compounds in self-administration assays.

While attributed to antagonist effects at S1R and S2R, the exact mechanisms underlying the anti-allodynic and antinociceptive efficacy of CM-304 and AZ-66 in various inducible modalities of pain warrant further study. Previous studies have suggested a role for other signaling and receptor targets. For instance, sigma receptor antagonism was found to enhance norepinephrine levels while reducing formalin-induced glutamate release in the spinal cord, as well as attenuate wind up responses in spinal cords sensitized to repetitive nociceptive stimulation (Romero et al., 2012; Vidal-Torres et al., 2014). The latter effect on central sensitization may be a critical component in treating chronic pain for novel therapeutics. Activation of spinal S1Rs has also been reported to enhance NMDA receptor induced pain *via* a PKC/PKA-dependent phosphorylation of NR1 subunit on the NMDA receptor in male ICR mice. Interestingly, sigma agonists only potentiated pain when the NMDA system was activated by nociception (Kim et al., 2008; Yoon et al., 2010). Inhibitors of phospholipase C (PLC), PKC, and Ca^2+^-ATPase attenuated the S1R mediated pain facilitation, implicating the involvement of these secondary pathways in sigma receptor activation and nociceptive signaling (Roh et al., 2008). Agonist induced hypersensitivity and increased phosphorylation of the NR1 subunit were also blocked by NOS inhibitors, reversing sigma agonist-induced increased nNOS activity. This effect was blocked when protein phosphatase calcineurin was applied, but not soluble guanylyl cyclase (sGC; Roh et al., 2011). In sum, these signaling mechanisms combine to increase levels of intracellular calcium and activity of the phospholipase C-IP_3_ signaling cascades, potentially promoting nociception when sigma receptors are recruited by noxious stimuli to the plasma membrane of nociceptive components (Ueda et al., 2001). While direct examination of these nociceptive mechanisms was beyond the scope of this initial characterization study, we anticipate the present selective sigma receptor antagonists will facilitate future studies to better evaluate these factors free of off-target or subtype-receptor interactions.

Patients who suffer from neuropathic pain tend to require escalating treatment and report less effective pain relief (Torrance et al., 2007). Given the increasing side effects of clinically used opioids with increased dosage to compensate for limited efficacy, they are now considered second-line treatment for neuropathic pain. Calcium channel α2-δ ligands such as gabapentin are now considered a first-line treatment (O’Connor and Dworkin, 2009). However, these compounds also demonstrate adverse effects. Gabapentin is known to produce sedation, dizziness, and more importantly peripheral edema in patients, with renal insufficiency a major precaution when prescribing. Gabapentin given to elderly patients was also observed to cause or exacerbate cognitive or gait impairment (Calandre et al., 2016; Mangram et al., 2016). Several weeks may also be required to determine the effective dose, and evidence suggests that gabapentin is ineffective when treating chemotherapy induced neuropathic pain, indicating a need for alternative therapies (Wong et al., 2005; O’Connor and Dworkin, 2009). As suggested by the current data, S1R-selective antagonists such as CM-304 may provide analgesia and anti-allodynia with fewer liabilities of use. Supporting this concept, it is notable that the radioligand analog of CM-304, FTC-146 (James et al., 2012; James et al., 2014), has been successful and well tolerated in phase I clinical trials to image S1Rs upregulated at the site of neuropathic injury with good safety indications (Shen et al., 2017a; Shen et al., 2017b). Taken together, these data suggest highly selective, metabolically stable S1R antagonists hold promise as novel non-opioid therapeutics for chronic pain management in complex patients.

Beyond the direct anti-allodynic effects of the sigma receptor antagonists, it is feasible they have value as adjuvants for pain management. The S1R antagonist E52862 was shown to potentiate morphine induced antinociception while also producing antinociception in morphine-tolerant mice (Vidal-Torres et al., 2013). Enhancement of morphine analgesia did not coincide with enhancement of other opioid effects, such as physical dependence, inhibition of GI transit, or mydriasis (Vidal-Torres et al., 2013). It is conceivable that adjuvant use of S1R antagonists co-administered with lower doses of opioids may produce adequate pain management without the adverse effects associated with elevated doses of opioids. The effects of CM-304 and AZ-66 on opioid-mediated analgesia and side effects such as antinociceptive tolerance were not evaluated in the present study, but warrant future study.

## Conclusion

The current findings support the development of sigma receptor antagonists as emerging novel therapeutics for the treatment of multiple modalities of pain.

## Data Availability Statement

The datasets generated for this study are available on request to the corresponding author.

## Ethics Statement

This study was carried out in accordance with the recommendations of the 2011 NIH Guide for the Care and Use of Laboratory Animals and ARRIVE guidelines overseen by the Institutional Animal Care and Use Committee at the University of Florida. The protocols (#201609530 and 201710031) were approved by the Institutional Animal Care and Use Committee at the University of Florida.

## Author Contributions

CM and JPM participated in research design. TC, SE, JMM, and LW conducted the experiments. MM and SI contributed new reagents or analytic tools. TC, LW, and JPM performed data analysis. TC, LW, CM, and JPM wrote and contributed to the writing of the manuscript.

## Conflict of Interest Statement

The authors declare that the research was conducted in the absence of any commercial or financial relationships that could be construed as a potential conflict of interest.
