# Supplementary material for: Characterization of Sigma 1 Receptor Antagonist CM-304 and Its Analog, AZ-66: Novel Therapeutics Against Allodynia and Induced Pain
Source: Front Pharmacol. 2019 Jun 14;10:678. doi: 10.3389/fphar.2019.00678 (PMC6586922; doi:10.3389/fphar.2019.00678)
Supplement: Supplementary file 1 [file DataSheet_1.pdf]

Cirino et al., Frontiers #457596: Supplemental material:

Supplemental Figure 1. Dose- and time-dependent ambulatory effects of i.p. administration of (**A**) CM-304 or (**B**) AZ-66 evaluated in the CLAMS assay with C57BL/6J mice. *=significantly different from vehicle effect (dashed line), p<0.05; two-way ANOVA w/ Dunnett's *post hoc* test. N=7-17 mice/group. ***Results expressed as % matching vehicle response***.

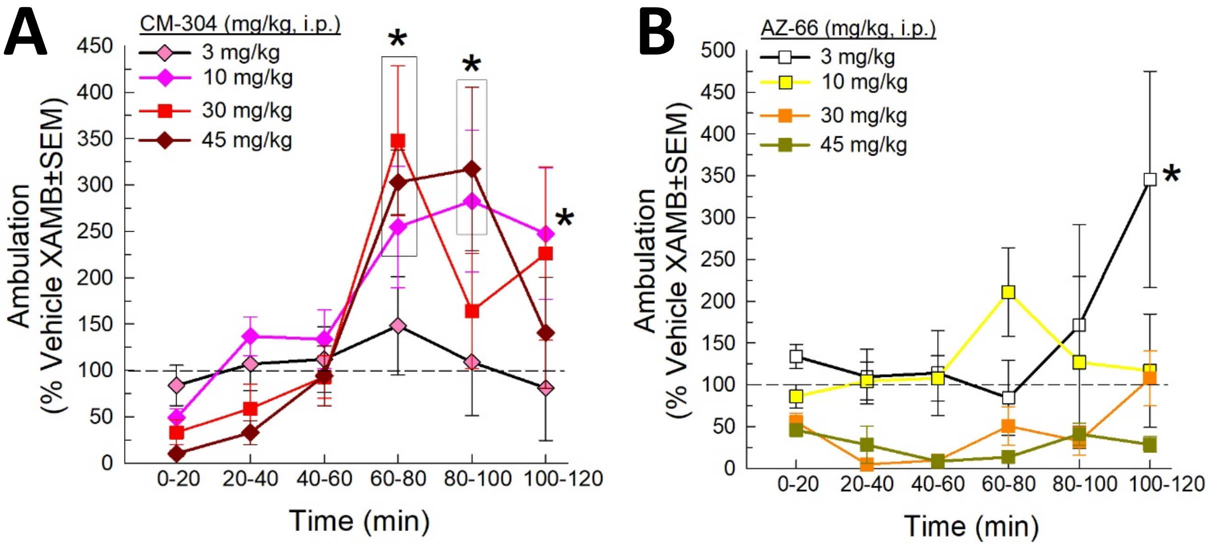

Cirino et al., Frontiers #457596: Supplemental material:

Supplemental Figure 2. Dose- and time-dependent ambulatory effects of i.p. administration of (**A**) Morphine and U50,488, (**B**) CM-304 or (**C**) AZ-66 evaluated in the CLAMS assay with C57BL6/J mice. *=significantly different from vehicle effect (dashed line), $p<0.05$; two-way ANOVA w/ Dunnett's *post hoc* test. N=16-20 mice/group for morphine and U50,488, and 7-17 mice/group for CM-304 and AZ-66 testing. ***Results are presented as raw ambulatory response, with a separate response of vehicle.***

Two-way ANOVA results (treatment x time):

Morphine: $F_{(10,270)}=10.6$, $p<0.0001$

U50,488: $F_{(10,258)}=9.84$, $p<0.0001$

CM-304: $F_{(20,345)}=5.72$, $p<0.001$

AZ-66: $F_{(20,265)}=1.76$, $p=0.02$

*p<0.05 or vs. time-matched vehicle response,

Dunnett's multiple comparisons *post hoc* test

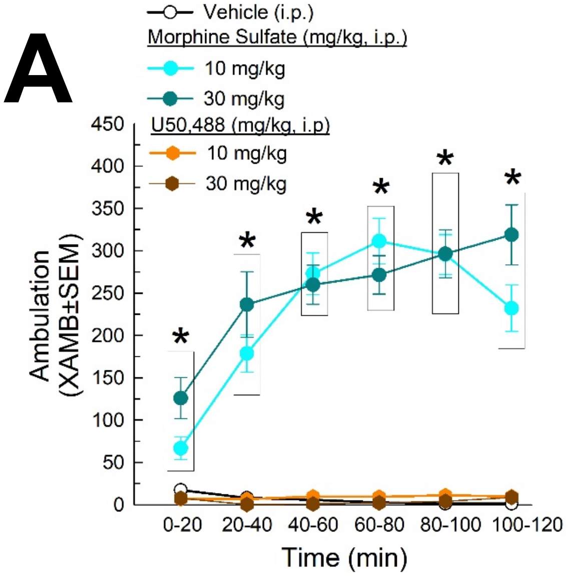

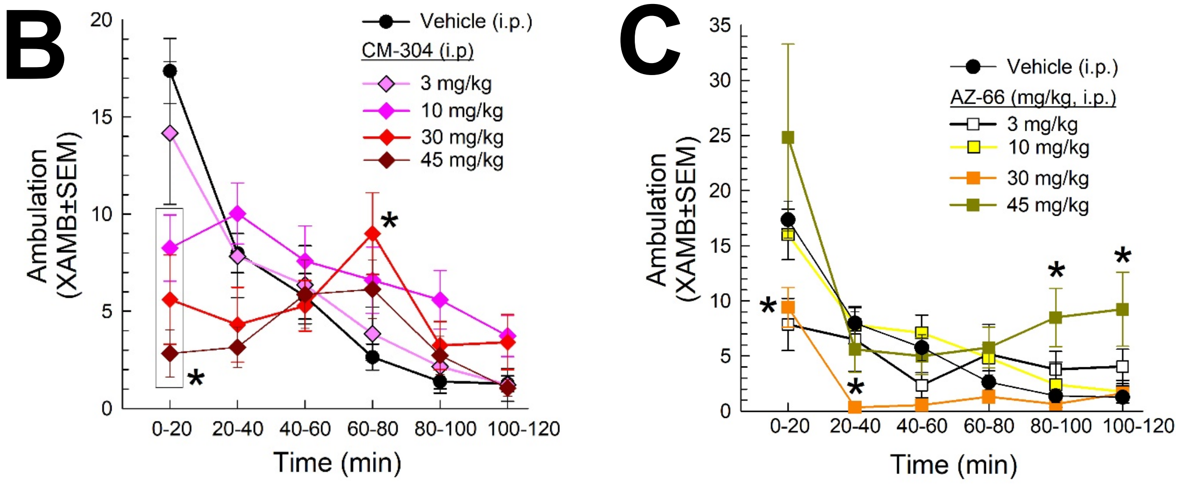

Supplemental Table 1: Raw place conditioning data collected for CM-304:

C57BL/6J mice: untreated prior to conditioning

CM304 (45 mg/kg, i.p.): 2 day counterbalanced **place-conditioning design.**

Mouse	Wt	Date	Free-Range Response (AM, no treatment yet)				Treatment (C	Free-Range Response (AM):			
No:	(g)	Start:	hicle Box time	304 Box time	between time	304-Veh Box	(AM) & Box	hicle Box time	304 Box time	between time	304-Veh Box Diff:
1	N/A	11/17/2014	395	752	653	357	Box 2-R	mouse died during injection			
2		11/17/2014	658	730	412	72	Box 2-R	544	567	689	23
3		11/17/2014	612	489	699	-123	Box 2-R	576	261	963	-315
4		11/17/2014	424	599	777	175	Box 2-R	449	616	735	167
5		11/17/2014	667	357	776	-310	Box 2-R	508	485	807	-23
6		11/17/2014	692	491	617	-201	Box 2-R	586	735	479	149
7		11/17/2014	422	763	615	341	Box 2-R	676	482	642	-194
8		11/17/2014	625	552	623	-73	Box 2-R	653	488	659	-165
9		11/17/2014	629	562	609	-67	Box 1-L	761	464	575	-297
10		11/17/2014	527	591	682	64	Box 1-L	324	682	794	358
11		11/17/2014	530	398	872	-132	Box 1-L	378	391	1031	13
12		11/17/2014	573	415	812	-158	Box 1-L	378	468	954	90
13		11/17/2014	293	675	832	382	Box 1-L	197	876	727	679
14		11/17/2014	543	571	686	28	Box 1-L	mouse died during injection			
15		11/17/2014	514	508	778	-6	Box 1-L	578	389	833	-189
16		11/17/2014	467	538	795	71	Box 1-L	513	275	1012	-238
17		11/17/2014	594	516	690	-78	Box 1-L	644	510	646	-134
18		11/17/2014	696	566	538	-130	Box 1-L	1038	304	458	-734
19		11/17/2014	409	601	790	192	Box 1-L	409	557	834	148
20		11/17/2014	486	525	789	39	Box 1-L	349	278	1173	-71
21	N/A	11/17/2017	773	528	499	-245	Box 1-L	1144	288	368	-856
22		11/17/2017	898	240	662	-658	Box 1-L	608	346	846	-262
23		11/17/2017	829	317	654	-512	Box 1-L	603	628	569	25
24		11/17/2017	800	484	516	-316	Box 1-L	601	627	572	26
25		11/17/2017	844	348	608	-496	Box 1-L	554	608	638	54
26		11/17/2017	636	467	697	-169	Box 1-L	484	624	692	140
27		11/17/2017	707	489	604	-218	Box 1-L	558	544	698	-14
28		11/17/2017	601	553	646	-48	Box 1-L	630	710	460	80
29		11/17/2017	697	601	502	-96	Box 2-R	480	605	715	125
30		11/17/2017	375	891	534	516	Box 2-R	751	472	577	-279
31		11/17/2017	561	697	542	136	Box 2-R	693	696	411	3
32		11/17/2017	792	483	525	-309	Box 2-R	645	333	822	-312
33		11/17/2017	630	527	643	-103	Box 2-R	497	327	976	-170
34		11/17/2017	480	513	807	33	Box 2-R	369	330	1101	-39
35		11/17/2017	452	577	771	125	Box 2-R	343	470	987	127
36		11/17/2017	592	565	643	-27	Box 2-R	810	542	448	-268
			595.08	541.08	663.83	-54		568.56	499.35	732.09	-69.21
			144.73	129.04	112.05	250.29		191.55	154.48	209	278.26
			24.12	21.51	18.68	41.72		32.85	26.49	35.84	47.72
			36	36	36	36		34	34	34	34

Explanation of the sample size for this experiment: Two (of 20) mice were lost due to unrelated operator error in making injections in the first test performed in 2014, so these mice were not used in the final analysis (as denoted by the grey highlighting). As initial CM-304 results with these 18 mice failed to show a clear result from the place conditioning tests (Pre-CPP: 3.22+43.24 s vs Post-CPP response of -40.72+71.1 s), whereas AZ-66 showed significant place aversion with 19 mice (losing one of the original 20 due to an unrelated injury), we added 16 more mice to the CM-304 testing to be certain of the absence of a CPP or CPA. As can be seen from the raw data presented here, this additional data did not generate a significantly different result of the original test. To be fully transparent, all data was presented as the final data set.
